# The impact of COVID-19 on an Irish Emergency Department (ED): a cross-sectional study exploring the factors influencing ED utilisation prior to and during the pandemic from the patient perspective

**DOI:** 10.1186/s12873-022-00720-7

**Published:** 2022-11-02

**Authors:** Niamh M. Cummins, Carrie Garavan, Louise A. Barry, Collette Devlin, Gillian Corey, Fergal Cummins, Damien Ryan, Gerard McCarthy, Rose Galvin

**Affiliations:** 1 School of Medicine, Faculty of Education and Health Sciences, University of Limerick, Limerick, Ireland; 2 SLÁINTE Research and Education Alliance in General Practice, Primary Healthcare and Public Health, Faculty of Education and Health Sciences, University of Limerick, Limerick, Ireland; 3 Ageing Research Centre, Health Research Institute, University of Limerick, Limerick, Ireland; 4 Department of Paramedicine, Monash University, Melbourne, Australia; 5 Present Address: Department of Nursing and Midwifery, Faculty of Education and Health Sciences, University of Limerick, Limerick, Ireland; 6 School of Allied Health, Faculty of Education and Health Sciences, University of Limerick, Limerick, Ireland; 7 ALERT Limerick EM Education Research Training, Emergency Department, University Hospital Limerick, Limerick, Ireland; 8 Emergency Department, Cork University Hospital, Cork, Ireland

**Keywords:** Emergency department utilisation, Crowding, COVID-19, Access to care, Emergency department operations

## Abstract

**Background:**

The collateral damage of SARS-CoV-2 is a serious concern in the Emergency Medicine (EM) community, specifically in relation to delayed care increasing morbidity and mortality in attendances unrelated to COVID-19. The objectives of this study are to describe the profile of patients attending an Irish ED prior to, and during the pandemic, and to investigate the factors influencing ED utilisation in this cohort.

**Methods:**

This was a cross-sectional study with recruitment at three time-points prior to the onset of COVID-19 in December 2019 (*n* = 47) and February 2020 (*n* = 57) and post-Lockdown 1 in July 2020 (*n* = 70). At each time-point all adults presenting over a 24 h period were eligible for inclusion. Clinical data were collected via electronic records and a questionnaire provided information on demographics, healthcare utilisation, service awareness and factors influencing the decision to attend the ED. Data analysis was performed in SPSS and included descriptive and inferential statistics.

**Results:**

The demographic and clinical profile of patients across time-points was comparable in terms of age (*p* = 0.904), gender (*p* = 0.584) and presenting complaint (*p* = 0.556). Median length of stay in the ED decreased from 7.25 h (IQR 4.18–11.22) in February to 3.86 h (IQR 0.41–9.14) in July (*p* ≤ 0.005) and differences were observed in disposition (*p* ≤ 0.001). COVID-19 influenced decision to attend the ED for 31% of patients with 9% delaying presentation. Post-lockdown, patients were less likely to attend the ED for reassurance (*p* ≤ 0.005), for a second opinion (*p* ≤ 0.005) or to see a specialist (*p* ≤ 0.05).

**Conclusions:**

Demographic and clinical presentations of ED patients prior to the first COVID-19 lockdown and during the reopening phase were comparable, however, COVID-19 significantly impacted health-seeking behaviour and operational metrics in the ED at this phase of the pandemic. These findings provide useful information for hospitals with regard to pandemic preparedness and also have wider implications for planning of future health service delivery.

**Supplementary Information:**

The online version contains supplementary material available at 10.1186/s12873-022-00720-7.

## Background

ED crowding is a global public health crisis resulting in adverse outcomes for patients, communities and health services [1]. It is a significant patient safety issue associated with poor quality of care and increased mortality rates [2]. The causes of ED crowding involve a complex network of interwoven processes ranging from viral epidemics to hospital workflow [3].

In December 2019, a novel coronavirus disease (COVID-19) caused by severe acute respiratory syndrome coronavirus 2 (SARS-CoV-2) emerged in Wuhan, China [4]. The first imported cases of COVID-19 and community transmission were reported in Ireland in late February 2020 [5]. On 11th March, 2020 the World Health Organisation (WHO) declared the COVID-19 outbreak as a pandemic [6] and a “lockdown” commenced in Ireland on 27th March 2020. In the United States the WHO announcement coincided with a significant decrease in ED visits that week following months of relative stability [7].

As COVID-19 cases increased worldwide, emerging data suggested a decline in patients seeking emergency medical care for other illnesses [8, 9]. In Ireland, a paediatric ED reported a reduction of almost 50% in attendances during the pandemic compared with the same time-period for the preceding 2 years [10]. This indicates that some patients opted for an alternative pathway for medical care than the ED while others may have postponed or forgone care altogether.

Indirect effects of lockdown including a reduction of working hours and accidents contributed to the decline in ED attendance [11]. Hospital admissions for trauma reportedly decreased with motor vehicle collisions and fall-related injuries declining significantly in the early months of the pandemic [12]. However, there was also a consensus among clinicians that patients avoided seeking medical attention in the ED due to fear of infection [13]. This raises concerns about delayed care leading to increased morbidity and mortality in conditions such as stroke [14] and myocardial infarction [8] and the collateral damage of COVID-19. Giannouchos et al. described ED visits for emergent conditions including collapse, syncope, tachycardia, palpitations and open wounds as being similar to 2019 during the first wave of the pandemic in 2020 [15]. Other studies reported that triage level 1 remained unchanged meaning that the sickest patients still presented to the ED but that the distribution of acuity was altered [10, 16]. It’s possible that COVID-19 may have served to highlight the overuse of EDs by non-emergency and low acuity patients that could be managed more appropriately in other settings.

A combination of physiological, psychological and social factors impact the decisions of patients to present to the ED. It is important to track and understand trends in EM and this is particularly significant with the increased complexity of care arising from SARS-CoV-2. The objectives of this study are to describe the impact of COVID-19 on an Irish ED and to explore the factors influencing ED attendance before and during the pandemic from the patient perspective.

## Methods

### Aim

The primary aim of this study is to describe changes in the demographic and clinical profile of patients attending an Irish ED in the months preceding COVID-19 and during the pandemic. A secondary aim is to investigate the factors influencing ED utilisation in this cohort.

### Setting

The setting for this study was the ED at University Hospital Limerick (UHL), a University Teaching hospital in the Mid-West of Ireland. UHL serves an urban and rural catchment population of 473,000 in Limerick city and the counties of Limerick, Clare and North Tipperary. In 2019 preceding the COVID-19 pandemic there were 71,315 presentations to the ED, averaging 195/day [17] and a further 33,069 presentations at the three affiliated Injury Units in the region.

### Design

This study was conducted in collaboration with a larger census project (Better Data, Better Planning; The BDBP Study) which profiled ED attendance in selected hospitals nationally [18]. It was a cross-sectional study profiling change in attendance at a single ED at multiple time-points prior to and during the pandemic. Data were collected at UHL over a 24-hour period in Winter and Summer to account for diurnal and seasonal variation in attendance patterns. The sampling frame for each time-point was Thursday at 12 pm - Friday at 12 pm. Data collection occurred at three time-points; December 2019, February 2020 and July 2020. “Lockdown” commenced in Ireland on 27th March 2020 and concluded on 18th May 2020 with a phased reopening [19]. By July 2020 under “Phase 3”, most retail was operating normally, however, the service industry was restricted and limited travel was permitted.

### Participants and procedure

All adult patients attending the ED over the sampling frame were potentially eligible for recruitment. The Manchester Triage System (MTS) is used in most Irish and UK EDs to assess the degree of severity of cases, based on presenting signs and symptoms. It assigns an order of clinical priority by allocating patients to one of five urgency categories (Immediate, Very Urgent, Urgent, Standard and Non-urgent) which determine safe waiting times in the ED [20]. The following inclusion criteria were applied in this study A) Adult aged ≥18 years B) Medically stable MTS categories 2–5 C) Patient has capacity and willingness to provide informed consent. Exclusion criteria include; A) Scheduled admissions to the ED B) Mental Health presentations C) Patients with altered capacity due to drug or alcohol intoxication D) Inability to communicate sufficiently in English to participate. The Triage Nurse acted as the Study Gatekeeper and following consultation with the wider Multidisciplinary Team, the Research Nurses determined when the participant could be recruited without impacting on any treatment or diagnostics they were receiving. Patients who were initially deemed unable to participate due to e.g. pain, nausea or distress were re-assessed once they had received treatment. All patients meeting the criteria and deemed well enough to participate were invited to enrol in the study and served as the study denominator, with the final sample size dependant on the number of patients consenting to participate.

Written informed consent was obtained by the Research Nurses, participants then completed a self-report questionnaire and provided consent for access to medical charts including; demographics, public/private health insurance, socioeconomic status, presenting complaint, triage category, length of stay and disposition. The questionnaire was developed by a multidisciplinary team of EM clinicians in collaboration with the researchers, it was externally reviewed, piloted internally and questions were refined based on feedback prior to full implementation. The questionnaire design incorporated open-ended questions, rating scales and multiple-choice questions. For multiple-choice questions, all responses were included in the analysis and percentage respondents was reported. The questionnaire explored the following categories; demographics, healthcare utilisation, service awareness and factors influencing decision to attend the ED. Demographic variables included marital status, living arrangements, education level and occupational status.. Socioeconomic status was recorded by electoral division as these are the smallest legally defined administrative areas in the State for which Small Area Population Statistics (SAPS) are published from the Census [20]. Proximity to health services (i.e. distance to GP and ED in kilometres from home address) was self-reported by participants.

Data was collected on the duration of presenting complaint and community services accessed prior to attendance. Utilisation of healthcare services in the past year was documented including hospital services (out-patient appointment, ED, hospital admission) and community services (General Practitioner; GP, Public Health Nurse; PHN and other Allied Health Professionals. Awareness of alternative services for emergency care including Injury Units and out-of-hours GP (OOH-GP) were also recorded. The questionnaire explored reasons for ED attendance, including self-assessment of pain and level of concern regarding presenting complaint, on a numeric rating scale [1–10].

During data collection in July 2020 (post-lockdown) local infection control policies and protocols were adhered to by the research team. The questionnaire was also updated to evaluate outcomes specifically related to COVID-19. Participants were asked about access to health services during the pandemic, whether COVID-19 influenced their decision to attend the ED and their level of concern regarding attendance. Additionally new protocols had also been implemented during lockdown with regard to triage, which were in effect in July. Patients screening positive for COVID-19 symptoms, trauma presentations, patients requiring resuscitation and those suitable for Clinical Decision Unit (CDU) assessment were treated in the ED. All other patients were referred to the Medical Assessment Unit (MAU) or the Surgical Assessment Unit (SAU) for treatment, and both units were functioning at increased capacity with additional staffing and operational hours.

### Patient and public involvement

Direct patient and public consultation was not undertaken, however this research was informed by previous studies detailing service user experiences of emergency care in Ireland [21, 22].

### Data analysis

Data extraction was performed by the Research Nurse with data entry and coding being conducted in Excel (Microsoft, San Diego, CA), followed by analysis in SPSS (IBM SPSS Statistics Version 26, Armonk, NY). Normality testing of variables was performed using the Kolmogorov–Smirnov test. Categorical data are presented as frequencies and percentages and continuous variables are presented as mean (standard deviation; SD) or median (Interquartile Range; IQR), depending on normality. The chi-square test was utilised to examine relationships between categorical variables. For group comparisons, ANOVA or the Kruskall-Wallis test was utilised, including post hoc Mann-Whitney U tests. A value of *p* < 0.05 was considered to be statistically significant.

## Results

### Study population and demographics

**Fig. 1:**
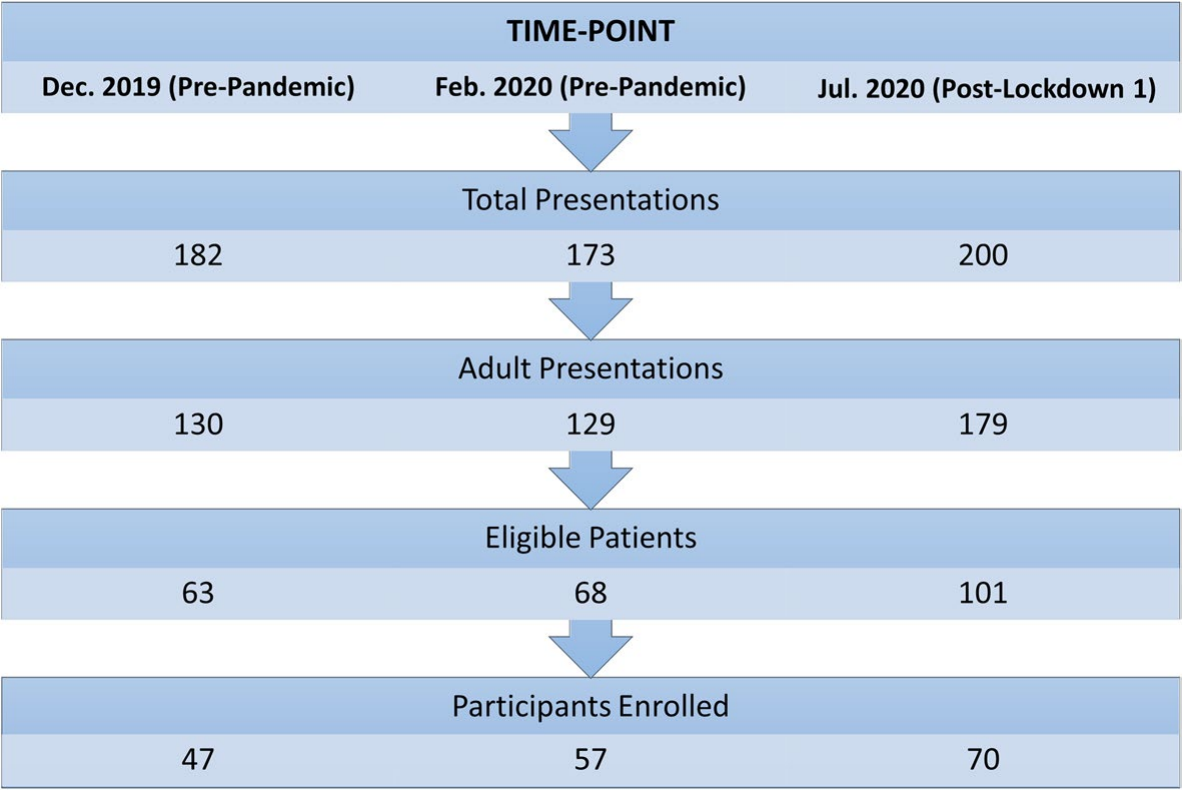
Participant screening and enrollment for each time-point

Patients meeting the inclusion criteria comprised the study denominator (Fig. 1) and the proportion of eligible participants enrolled in December was 47/63 (75%), in February was 57/68 (84%) and in July was 70/101 (69%). Participants were demographically very similar at each time-point and there were no significant differences in characteristics between groups (Table 1).

**Table 1:** Sociodemographic characteristics of study participants (*n* = 174)

Category	Variable	TOTAL (***n*** = 174)	DEC (***n*** = 47)	FEB (***n*** = 57)	JUL (***n*** = 70)	***P*** value
**Gender**	Female	83, 48%	24, 51%	24, 42%	35, 50%	0.584
	Male	91, 52%	23, 49%	33, 58%	35, 50%	
**Age**	Mean ± SD	52 ± 19	53 ± 19	51 ± 21	51 ± 18	0.904
	Range	18–91	19–86	18–91	20–85	
	18-39y	55, 32%	14, 30%	21, 37%	20, 29%	
	40-64y	69, 40%	18, 38%	21, 37%	30, 43%	
	65y+	50, 29%	15, 32%	15, 26%	20, 29%	
**Civil Status**	Partner	17, 10%	6, 13%	5, 9%	6, 9%	0.804
	Married	79, 45%	22, 47%	26, 46%	31, 44%	
	Separated/Divorced	19, 11%	5, 10%	4, 7%	10, 14%	
	Widowed	10, 6%	4, 9%	2, 4%	4, 6%	
	Single	48, 28%	10, 21%	19, 34%	19, 27%	
**Residential Status**	Partner	82, 47%	25, 53%	26, 46%	31, 44%	0.836
	Family	47, 27%	12, 26%	14, 25%	21, 30%	
	Lives Alone	31, 18%	8, 17%	9, 16%	14, 20%	
	Other e.g. co-share	13, 7%	2, 4%	7, 13%	13, 6%	
**Principal Status**	Employee	68, 39%	20, 43%	24, 42%	24, 35%	0.729
	Self-employed	8, 5%	2, 4%	4, 7%	2, 3%	
	Work in the Home	19, 11%	5, 11%	5, 9%	9, 13%	
	Retired	50, 29%	15, 32%	14, 25%	21, 31%	
	Unemployed	22, 13%	5, 11%	7, 12%	10, 15%	
	Student	5, 3%	0, 0%	3, 5%	2, 3%	
**Education**	No formal education	5, 3%	3, 6%	1, 2%	1, 1%	0.608
	Primary	20, 12%	3, 6%	7, 13%	10, 14%	
	Secondary	75, 43%	23, 49%	27, 38%	25, 36%	
	Technical/Vocational	26, 15%	7, 15%	7, 13%	12, 17%	
	Third Level	47, 27%	11, 23%	14, 25%	22, 31%	
**Socioeconomic Status**	Affluent	1, 1%	0, 0%	0, 0%	1, 1%	0.332
	Above Average	73, 42%	17, 38%	25, 45%	31, 45%	
	Below Average	66, 38%	18, 40%	18, 32%	30, 44%	
	Disadvantaged	21, 12%	5, 11%	12, 21%	4, 6%	
	Very Disadvantaged	9, 5%	5, 11%	1, 2%	3, 4%	
**Healthcare Coverage**	Private Insurance	69, 40%	20, 44%	20, 36%	29, 41%	0.593
	Public - Medical Card	72, 41%	19, 41%	21, 38%	32, 46%	
	Public - No Cover	31, 18%	7, 15%	15, 27%	9, 13%	
**Distance to GP**	Median (IQR)	3 (1–10)	4 (1–11)	3 (1–7)	3 (1–10)	0.531
	Range	1–35	1–35	1–30	1–28	
	< 5 km	98, 56%	25, 53%	34, 61%	39, 57%	
	< 10 km	31, 18%	9, 19%	10, 18%	12, 17%	
	< 15 km	23, 13%	4, 9%	8, 14%	11, 16%	
	≥15 km	20, 12%	9, 20%	4, 7%	7, 10%	
**Distance to ED**	Median (IQR)	21 (7–38)	21 (7–35)	21 (5–31)	28 (10–40)	0.219
	Range	1–140	1–84	1–70	1–140	
	< 10 km	50, 29%	16, 34%	19, 33%	15, 22%	
	< 25 km	39, 23%	10, 21%	12, 21%	17, 25%	
	< 50 km	61, 35%	14, 30%	20, 35%	27, 39%	
	≥50 km	23, 13%	7, 15%	6, 11%	10, 14%	
**Mode of Transport to ED**	Ambulance	29, 17%	5, 11%	12, 21%	12, 17%	0.159
	Private Car	121, 70%	35, 75%	37, 65%	49, 70%	
	Public Transport	19, 10%	5, 11%	5, 9%	9, 13%	
	Walk	5, 3%	2, 4%	3, 5%	0, 0%	

^a^Individual cases of missing data were excluded from analysis, this occurred in a small number of cases E.g. Data was not traceable on hospital systems or in the event a participant chose not to respond to a question on the survey

### Factors influencing decision to attend ED

#### Referral from community services

Prior to ED attendance 68% of all participants in the study (*n* = 174) accessed some form of community services (e.g. GP, Walk-in Clinic, PHN, Occupational Health, Pharmacist, Optician) with no significant difference between groups (December 70%, February 74%, July 61%; *p* = 0.312). GP services were accessed most frequently (59%) at each timepoint (December 51%, February 63%, July 61%; *p* = 0.421).

Participants were asked about their decision regarding GP consultation prior to ED attendance (Supplementary Table S1). The most frequent response was “Yes, I saw my GP and was told to go to the ED” (December 45%, February 57%, July 50%; *p* = 0.504). GP telephone consultations increased across time-points but did not reach significance (December 6%, February 11%, July 19%; *p* = 0.127). Participants who did not consult a GP frequently reported that “my problem is best dealt with in the ED” (December 17%, February 20%) although this decreased to 7% in July (*p* = 0.107).

Participants were also asked about utilisation of Community Health services over the past year (Supplementary Table S2). There were no significant differences observed between groups with regard to health services utilisation. However, utilisation of Psychology or Counselling Services was reported by 2% in December, 0% in February and 7% in July which did approach significance (*p* = 0.08). Utilisation of GP services over the past year did not differ across time-points (*p* = 0.429) A frequency of ≥7 GP visits per year was reported by 30, 19 and 19% in December, February and July respectively.

#### Alternative care pathways for emergencies – awareness and utilisation

Participants were asked about awareness of alternative pathways for emergency care, defined specifically by the ability to name local services including Injury Units and OOH-GP. Awareness of Injury Units in the full cohort was 31% (December 38%, February 35%, July 21%; *p* = 0.099) and awareness of OOH-GP services was 78% (December 85%, February 67%, July 83%; *p* ≤ 0.05). Participants were also asked about utilisation of alternative care pathways over the previous year. (Supplementary Table S2). There was no significant differences between groups over the past year with the Injury Unit being attended by 8% in December, compared to 12% for February and 6% for July (*p* = 0.436) and OOH-GP utilisation was 36, 25 and 25% respectively (*p* = 0.329).

#### Reasons for ED attendance

In terms of general reasons for attending the ED, most participants believed it was the “best place” (December 63%, February 79%, July 77%). Participants were also unaware of other services available for treatment (December 37%, February 28%, July 35%) or were unaware of other services open at the time (9, 4, 14%, respectively). In July 18% of participants reported that their reason for attendance was related to COVID-19 restrictions (Table 2).

**Table 2:** General and specific reasons for attendance at the ED from the patient perspective (*n* = 174) ^a^

General Reasons for ED Attendance	TOTAL	DEC	FEB	JUL	***P*** value
The ED is the best place for my problem	70%	63%	79%	77%	0.186
I’m unaware of other services to treat me for this problem	33%	37%	28%	35%	0.615
My family told me to come to the ED	13%	15%	14%	11%	0.706
I don’t know what other services are open at this time	9%	9%	4%	14%	0.160
It is easy for me to get to the ED	4%	2%	4%	6%	0.596
I attended the ED before and I was happy with it	4%	0%	4%	8%	0.146
I think I will be seen quicker here than at any other service	3%	4%	2%	3%	0.740
I usually come to ED with a medical problem	2%	0%	2%	3%	0.502
I could not afford to go anywhere else	1%	0%	2%	0%	0.366
With COVID-19 restrictions the ED is where I needed to attend ^*b*^	–	–	–	18%	–
**Specific Reasons for ED Attendance**	**TOTAL**	**DEC**	**FEB**	**JUL**	***P*** **value**
I consider this condition to be an emergency	47%	45%	54%	43%	0.354
I thought I needed an x-ray or scan	24%	23%	33%	17%	0.089
I thought I might need to go into hospital	23%	28%	26%	17%	0.275
I need reassurance that my illness/injury is not serious	21%	23%	33%	9%	≤0.005
I came to the ED to get a second opinion	14%	15%	25%	3%	≤0.005
I wanted to see a specialist	12%	17%	18%	3%	≤0.05
I wanted to see a doctor or a nurse as soon as possible	12%	13%	19%	6%	0.078
I thought I needed the wound treated	5%	4%	7%	5%	0.767
I thought I might need a blood test	5%	4%	9%	3%	0.333
I thought I might need a tetanus injection	1%	2%	0%	2%	0.575
I am on a waiting list and I thought this would speed it up ^*b*^	1%	2%	0%	0%	0.265
I could not attend my normal service due to COVID-19 restrictions	–	–	–	3%	–
Other Reason* E.g. GP Referral	29%	23%	19%	42%	≤0.05

^a^Individual cases of missing data were excluded from analysis, this occurred in a small number of cases E.g. Data was not traceable on hospital systems or in the event a participant chose not to respond to a question on the survey

^b^Similar questionnaire responses were combined for the purposes of analysis (e.g. suspected fracture/X-ray required)

With regard to specific reasons for attendance, most patients considered their condition to be an emergency (December 45%, February 54%, July 43%). Participants frequently thought that an x-ray or scan was required (December 23%, February 33%, July 17%) or that hospital admission may be necessary (December 28%, February 26%, July 17%). In comparison to December and February, patients in July were less likely to attend the ED for reassurance (23, 33, 9%; *p* ≤ 0.005), to obtain a second opinion (15, 25, 3%; *p* ≤ 0.005) or to see a specialist (17, 18, 3%; *p* ≤ 0.05).

### Clinical outcomes

Clinical presentations to the ED were similar at each time-point (Table 3). The most common Presenting Complaint descriptor was “Unwell Adult” in December (19%) and July (24%) with “Chest Pain” most common in February (21%). One third of participants in July (33%) reported a duration of presenting complaint of < 1 day while 45% of participants in December and 39% in February had symptoms for > 7 days (*p* = 0.327). Worry levels were highest in December (Median 8; IQR 5–10) compared to February (7; IQR 5–8; *p* ≤ 0.05) and July (6; IQR 3–8; *p* ≤ 0.001). Pain levels did not statistically differ between groups, although this did approach significance (*p* = 0.06). The median rating for pain in December was 7 (IQR 4–8) compared to 6 (IQR 3–8) in February and 5 (IQR 1–7) in July.

**Table 3:** Clinical characteristics of study participants (*n* = 174) ^a^

Category	Variable	TOTAL (***n*** = 174)	DEC (***n*** = 47)	FEB (***n*** = 57)	JUL (***n*** = 70)	***p*** value
**Presenting Complaint**	Abdominal Pain	20, 12%	5, 11%	10, 18%	5, 7%	*p* = 0.556
	Chest Pain	23, 13%	4, 9%	12, 21%	7, 10%	
	Limb Problem	22, 13%	6, 13%	9, 16%	7, 10%	
	Shortness of Breath	14, 8%	4, 9%	7, 12%	3, 4%	
	Unwell Adult	34, 20%	9, 19%	8, 14%	17, 24%	
	Wound	10, 6%	3, 6%	1, 2%	6, 9%	
	Other*	51, 28%	16, 33%	10, 17%	25, 36%	
**Duration of Complaint**	< 1 day	57, 33%	18, 38%	16, 28%	23, 33%	*p* = 0.327
	1–2 days	22, 13%	2, 4%	7, 12%	13, 19%	
	3–7 days	35, 20%	6, 13%	12, 21%	17, 24%	
	> 7 days	60, 35%	21, 45%	22, 39%	17, 24%	
**Worry Scale (1–10)**	Median	7	8	7	6	*p* ≤ 0.005
	IQR	5–8	5–10	5–8	3–8	
**Pain Scale (1–10)**	Median	6	7	6	5	*p* = 0.056
	IQR	2–8	4–8	3–8	1–7	
**Triage Category (MTS 2–5)**	Very Urgent (MTS 2)	26, 15%	2, 5%	16, 28%	8, 12%	*p* ≤ 0.05
	Urgent (MTS 3)	126, 74%	37, 84%	35, 61%	54, 78%	
	Standard (MTS 4)	17, 10%	5, 11%	5, 9%	7, 10%	
	Non-Urgent (MTS 5)	1, 1%	0, 0%	1, 2%	0, 0%	
**Length of Stay (LOS)**	Median	6.17	7.17	7.25	3.86	*p* ≤ 0.005
	IQR	2.39–10.11	3.39–9.59	4.18–11.22	0.41–9.14	
	Range	0.07–67.12	0.49–50.22	0.25–67.12	0.07–38.27	
	< 1 h	26, 16%	1, 2%	2, 4%	23, 33%	
	1-2 h	12, 7%	5, 11%	2, 4%	5, 7%	
	2-4 h	23, 13%	8, 17%	8, 14%	7, 10%	
	4-8 h	44, 25%	14, 30%	19, 33%	11, 16%	
	8-16 h	50, 29%	13, 28%	21, 37%	16, 23%	
	16-24 h	9, 5%	1, 2%	4, 7%	4, 6%	
	> 24 h	9, 5%	5, 11%	1, 2%	3, 4%	
**Disposition**	Admitted	43, 25%	12, 26%	19, 33%	12, 17%	*p* ≤ 0.001
	Discharged	123, 72%	31, 68%	34, 60%	57, 82%	
	- ED	97, 56%	31, 66%	32, 56%	34, 49%	
	- AMU^b^	13, 8%	1, 2%	2, 4%	10, 14%	
	- SAU^c^	13, 8%	0, 0%	0, 0%	13, 19%	
	Hospital Transfer	2, 1%	0, 0%	1, 2%	1, 1%	
	Did Not Wait	6, 3%	3, 6%	3, 5%	0, 0%	

^a^Individual cases of missing data were excluded from analysis, this occurred in a small number of cases E.g. Data was not traceable on hospital systems or in the event a participant chose not to respond to a question on the survey

^b^The Acute Medical Unit (AMU) assesses and treats acutely unwell medical patients who have been referred by a medical practitioner

^c^The Surgical Assessment Unit (SAU) provides a rapid access facility for acute surgical patients who have been assessed by a medical practitioner

With regard to triage category, in the overall cohort, chest pain comprised 19% of Very Urgent (Category 2) cases, unwell adults constitute the majority (21%) of Urgent cases (Category 3) while Limb problems (24%) and eye problems (24%) were most frequently triaged as standard (Category 4). Significant differences were observed between groups by triage category (*p* ≤ 0.05) with Very Urgent cases highest in February at 28% compared to December 5% and July 12%. Length of Stay (LOS) in the ED also differed significantly between groups (*p* ≤ 0.005). Median LOS in July was 3.86 h (IQR 0.41–9.14) in comparison to 7.25 h (IQR 4.18–11.22) in February and 7.17 h (IQR 3.39–9.59) in December. By LOS category, most participants stayed in the ED for < 1 hour in July (33%) compared to 4–8 hours in December (30%) and 8–16 hours in February (37%). In terms of disposition outcome most participants were discharged home following treatment in the ED (December 66%, February 56%, July 49%). Hospital admissions were highest in February at 33% compared to 26% in December and 17% in July (*p* = 0.07). Significant differences were observed between groups in relation to overall disposition (*p* ≤ 0.001) with increased referrals to the Acute Medical Unit (AMU) in July than in December or February (14%, vs. 2% vs. 4%) and to the Surgical Assessment Unit (SAU) at 19% vs. 0% vs. 0%.

Utilisation of the ED and other hospital services over the previous year were also recorded with no significant differences observed across time-points (Supplementary Table S2). ED attendances were reported by 49% in December, 54% in February and 59% in July (*p* = 0.705). Multiple visits [2–7] to the ED were recorded by 27, 25 and 29% respectively. Hospital admission(s) over the past year were also relatively common (December 28%, February 33%, July 41%; *p* = 0.262).

### Impact of COVID-19

Post-lockdown 31% of participants reported that COVID-19 influenced their decision to attend the ED. Patients reported feeling cautious about attending (26%) and weren’t sure what to expect in the ED (17%). Despite having symptoms, 9% of patients reported delaying their presentation to the ED due to concerns related to COVID-19. All of these patients were subsequently triaged as Urgent, suggesting an earlier presentation to the ED was likely warranted. Level of concern regarding COVID-19 in the ED was measured and the median value recorded was 3 (IQR 1–6) with 38% reporting no concerns (1/10) and just 5% reporting extreme concern (10/10). Subgroup analysis revealed no difference in concern levels by gender or age. Lockdown measures did not influence ability to travel to the ED with 97% reporting no transport issues. Accessibility of community health services was impacted by COVID-19, with 44% of patients reporting difficulties, most related to delay or postponement of services (33%). A total of 10% of participants reported that the GP was unable to provide care for their presenting complaint due to COVID-19 restrictions.

## Discussion

The demographic and clinical presentations of patients attending the ED prior to the first COVID-19 lockdown in Ireland and during the reopening phase were comparable, however, COVID-19 significantly impacted the health-seeking behaviour of patients and operational metrics in the ED at this phase of the pandemic.

In Ireland emergency public health measures for COVID-19 were directed by the National Public Health Emergency Team (NPHET). Capacity was created throughout the health service by cancellation of all elective procedures and non-urgent care in public hospitals and an agreement signed with private providers secured an additional 2000 acute beds and 47 critical care beds (operating essentially as public hospitals) [19]. While hospitals remained open during lockdowns restructuring of services locally did occur, for example one Dublin hospital transferred all paediatric services (including emergency care) to another location in order to safely maximise capacity for adult COVID-19 patients at the adult hospital which is co-located on site [23]. A National COVID-19 Telehealth Steering Committee was also established, and while some virtual clinics occurred in acute settings [24] telemedicine was implemented more widely in the community setting [25].

The collateral damage of SARS-CoV-2 has been a serious concern, specifically regarding delayed care increasing morbidity and mortality in ED attendances unrelated to COVID-19 [8, 14]. Anecdotal evidence regarding fear of infection may have been overestimated at this stage of the pandemic. The 14-day incidence rate for COVID-19 was low in July 2020 at 2.98 cases per 100,000 population and correspondingly the concern levels reported by patients in this study was relatively low (Median 3, IQR 1–6). However, delayed presentation to the ED related to COVID-19 concerns was reported by 9% of patients, all of whom were triaged as urgent. These attendances were potentially avoidable as delayed care usually results in more severe illness and higher risk of adverse outcomes [26]. In a Dutch study conducted during the first wave of the COVID-19 pandemic 20% of patients reported a delay in seeking emergency care [27]. Delayed care seeking behaviour was motivated by: fear of infection, reluctance to burden healthcare professionals, perceiving their own condition as less urgent relative to COVID-19 patients, and limited access to services, which is in agreement with the findings of this study.

In this study the pandemic influenced the decision to attend the ED in 31% of patients’ post-lockdown. Cancellation of elective and non-urgent care appeared to influence ED presentations with 44% of patients reporting difficulties in accessing services. Furthermore 10% of patients in this study presented to the ED as their GP was unable to provide care due to pandemic restrictions. Similar findings have been reported for other jurisdictions during the same time-period, in the United States 41% of adults reported forgone care from March through mid-July 2020, including general medical appointments, preventative care visits and elective surgery [28].

Healthcare utilisation in the past year did not differ across time-points but was relatively high suggesting that many patients in this cohort have underlying medical conditions. The presence of these underlying conditions and comorbidities may need addressing from a Public Health perspective in order to reduce the burden on EDs in the future. A recent systematic review found that an increase in presentations by patients with complex and chronic conditions is emerging as a significant driver of ED crowding [26]. With regard to utilisation of Psychology or Counselling Services over the past year, it is interesting to note that this was higher in the July cohort (7%) than in December (2%) or February (0%). This may suggest a negative impact of COVID-19 on the mental health of these participants, however it must be acknowledged that exact service access dates are unknown, the sample size is small and this result did not reach significance (*p* = 0.08).

A decrease in GP referrals to this ED had been observed earlier in the pandemic [23] but was not evident post-lockdown, although a non-significant increase in the proportion of GP tele-consultations was observed post-lockdown. Awareness of OOH-GP was high at 78% however awareness of Injury Units was much lower at 31%. Many patients in this study (24%) reported presenting to the ED for an x-ray or scan, and this suggests that improved awareness of Injury Units could potentially decrease ED attendance, which would be particularly important in the context of a pandemic.

In terms of other common reasons for ED attendance, ED patients frequently reported that their condition was an emergency and that the ED for the best place for treatment, which aligns with findings of previous studies [29, 30]. Reasons for attendance did differ across time-points, post-lockdown patients were significantly less likely to attend the ED for reassurance, to obtain a second opinion or to see a specialist. This suggests a possible shift in patient awareness of the critical nature of care in the ED but whether this will be sustained in the long-term cannot be determined.

Emergency hospital admissions were not significantly higher post-lockdown (*p* = 0.07) compared to pre-lockdown levels. This agrees with a time-series analysis in Scotland which revealed a drop in ED attendances and emergency admissions during lockdown, however by June these services were returning to historical levels [31]. In the current study workflow and particularly throughput in the ED remained significantly impacted by COVID-19 during the reopening phase of the pandemic. Median LOS in the ED decreased from 7.25 hours in February to 3.86 hours in July (*p* ≤ 0.005). This is also in agreement with findings from the UK where it was reported that the mean LOS for all ED attendances fell from 3.8 h to 3.1 h (*p* < 0.001) in the pandemic first wave period [32]. In our study decreased LOS in the ED mainly related to protocol changes in patient flow with higher utilisation of the AMU and SAU and, as a result of cancellation of elective care, reduced access block in the hospital to admissions. This finding is further supported by the Trolley Count in the ED in UHL which stood at 63 in February in comparison to 26 in July [33].

### Limitations

This research captures a snapshot of lower acuity attendances over a 24 h period at different time-points of the COVID-19 pandemic in 2020 at a single Irish ED, therefore findings may not be entirely generalisable to other settings. Presentations related to Mental Health or due to drug or alcohol intoxication were excluded from this study, due to issues around consent and interviewing a distressed patient. However, it must be acknowledged that these populations may be disproportionally affected by the COVID-19 pandemic. The questionnaire utilised in this study was developed within a larger census study on Irish ED and has not been externally validated. For patients reporting difficulties in accessing community services, it is difficult to ascertain if this related to an actual reduction in services or the perception of reduced access due to COVID-19 restrictions. Proximity to health services (GP and ED) were self-reported by patients and while this is acknowledged as being a crude measure, it was considered sufficient for the purposes of this study.

### Implications

Demographic and clinical presentations of ED patients prior to the first COVID-19 lockdown in Ireland and during the reopening phase were comparable, however, COVID-19 significantly impacted health-seeking behaviour and operational metrics in the ED at this phase of the pandemic. These findings provide useful information for hospitals with regard to pandemic preparedness and have wider implications for planning of future services in the ED and General Practice. Increased bed capacity in the hospital due to COVID-19 protocols meant that ED throughput was improved resulting in significantly reduced LOS for patients prior to admission. The results illustrate the importance of addressing capacity and flow in the wider hospital and within community services and may serve to highlight the fact that the solutions to ED crowding lie largely outside of the ED. The findings of this study indicate that sustainable system-wide solutions are required to tackle ED crowding, which if not addressed will remain a significant public health issue far beyond the COVID-19 pandemic.

## Supplementary Information

**Additional file 1.** Participant Questionnaire.

**Additional file 2: Table S1.** General Practitioner Consultation prior to Attendance at the ED (*n*=174) ^a^.

**Additional file 3: Table S2.** Health Service Utilisation in the last 12 months (*n*=174) ^a^.

## Data Availability

The datasets generated during the current study are not publicly available currently due to ongoing data analysis but are available from the corresponding author on reasonable request.

## Abbreviations

AMU: Acute Medical Unit
BDBP: Better Data, Better Planning
CDU: Clinical Decision Unit
ED: Emergency Department
EM: Emergency Medicine
GP: General Practitioner
MTS: Manchester Triage Score
OOH: Out-of-hours
OPD: Out-Patient Department
UHL: University Hospital Limerick
RN: Research Nurse
SAU: Surgical Assessment Unit
