# Supplementary material for: The impact of COVID-19 on an Irish Emergency Department (ED): a cross-sectional study exploring the factors influencing ED utilisation prior to and during the pandemic from the patient perspective
Source: BMC Emerg Med. 2022 Nov 2;22:176. doi: 10.1186/s12873-022-00720-7 (PMC9628103; doi:10.1186/s12873-022-00720-7)
Supplement: Supplementary file 1 — Additional file 1. Participant Questionnaire. [file 12873_2022_720_MOESM1_ESM.docx]

**Participant Questionnaire**

## **Section A: Your Current Visit**

**Q1** Describe the main problem that brings you to the Emergency Department (ED)?

______________________________________________________________________________________

**Q2** How long have you had this problem?

- 0-24 hours
- 1-2 days
- 2-7 days
- Longer than 1 week

**Q3** On a scale from 1 to 10, how worried (10 being most worried) would you say you are about the problem that brought you to the ED?

______________________________________________________________________________________

**Q4** On a scale from 1 to 10, how would you rate your pain level (10 being most severe) on arrival at the ED?

______________________________________________________________________________________

**Q5** Have you accessed any service in the community for this current problem prior to attending the ED today? YES/NO

If yes, please specify which service in the community?

- GP
- Public Health Nurse
- Walk-in clinic
- Pharmacy
- Occupational health department
- Other *(Please specify)* _________________________________________________________________
- Service not available due to COVID-19 restrictions *(Please specify)* ___________________________________________________________________________________

**Q6** Did you consider seeing your GP for this problem?

- Yes, but GP was unable to provide the treatment I needed at present due to COVID-19 restrictions
- Yes, I had a telephone consultation with my GP and was told to go to the ED
- Yes, I saw my GP and was told to go to the ED
- Yes, I saw my GP but was unhappy with the treatment
- Yes, I tried but could not contact my GP
- No, I thought that problem was best dealt with here in the ED
- No, I thought my GP would refer me to the ED
- No, I did not want to bother my GP
- No, the GP surgery was closed
- No, I am not happy with my current GP
- No, the GP is further away than the ED
- No, I do not have a GP
- No, some other reason *(Please specify)*_________________________________________________

**Q7** How did you get here today?

- Ambulance
- Private car
- Taxi
- Bus
- Walked
- Other *(Please specify)* ______________________________________________________________

**Q8** Did the COVID-19 restrictions impact on your ability to travel to the ED today? YES/NO

If Yes, then why?

__________________________________________________________________________________________

**Q9** Here are some of the reasons people give as to why they attend the ED for treatment. *Please select all that apply to your visit today*

- I am not aware of any other services to treat me for this current problem
- With the current COVID-19 restrictions the ED is where I had to/needed to attend.
- I don’t know what other services are open at this time
- The ED is the best place for my problem
- It is easy for me to get to the ED
- I usually come to ED with a medical problem
- I attended the ED before and I was happy with it
- I think I will be seen quicker here than at any other service
- My family told me to come to the ED
- I could not afford to go anywhere else

**Q10** What distance, to the nearest kilometre/mile is your home from the ED? ___________________________

**Q11** What distance, to the nearest kilometre/mile is your home from your family doctor? _________________

**Q12** Specifically regarding your current/presenting complaint *(Please select one option)*

- I consider this condition to be an emergency
- I thought I might need to go into hospital
- I don’t know whether it is broken or not
- I wanted to see a doctor or a nurse as soon as possible
- I need reassurance that my illness/injury is not serious
- I came to the ED to get a second opinion
- I thought I needed the wound treated
- I thought I might need a blood test
- I thought I needed an x-ray or scan
- I thought I might need a tetanus injection
- I wanted to see a specialist
- I am on a waiting list for an appointment for this problem and I thought it would speed it up
- I am on a waiting list for a test/investigation for this problem and thought it would speed it up
- Where I normally attend is not offering their service at present due to COVID-19 restrictions
- Other *(Please specify)* _________________________________________________________________

**Section B: Health Services**

**Q1** Healthcare Coverage

*Please circle YES/NO*

- Do you have private health insurance? YES/NO
- Do you have a medical card? YES/NO
- Do you have a GP visit card? YES/NO
- Do you have a long-term illness book? YES/NO

**Q2** In the last 12 months how many times have you seen your GP? *Please select one option*

- Not at all
- 1 time
- 2-3 times
- 4-6 times
- >6 times

**Q3** In the last 12 months how many times have you visited the ED? *Please select one option*

- Not at all
- 1 time
- 2-3 times
- 4-6 times
- >6 times

**Q4** In the last 12 months how many times have you been admitted to hospital? *Please select one option*

- Not at all
- 1 time
- 2-3 times
- 4-6 times
- >6 times

**Q5** In the last 12 months how many times have you visited an outpatient clinic? *Please select one option*

- Not at all
- 1 time
- 2-3 times
- 4-6 times
- >6 times

**Q6** In the last 12 months, how many times have you seen the public health nurse? *Please select one option*

- Not at all
- 1 time
- 2-3 times
- 4-6 times
- >6 times

**Q7** In the last 12 months, how many times have you seen an allied health professional (Physiotherapist, Occupational Therapist, Medical Social Worker, Speech Language Therapist)? *Please select one option*

- Not at all
- 1 time
- 2-3 times
- 4-6 times
- >6 times

## **Section C: Service Awareness**

**Q1** Injury Units are for the treatment of broken bones, dislocations, sprains, strains, wounds, scalds and minor burns that are unlikely to need hospital admission. Are you aware of a minor injury unit in your area? YES/NO

If yes, what is the name of this service? __________________________________________________________

If yes, in the last 12 months, how many times have you used this service? *Please select one option*

- Not at all
- 1 time
- 2-3 times
- 4-6 times
- >6 times

**Q2** Are you aware of a GP service that you can attend once your GP surgery closes in the evening and at the weekend? YES/NO

If yes, what is the name of this service? __________________________________________________________

If yes, in the last 12 months, how many times have you used this out-of-hours service? *Please select one option*

- Not at all
- 1 time
- 2-3 times
- 4-6 times
- >6 times

**Q3** Have you used any of the following services in the last 12 months? *Please select all that apply*

- Day case procedure or investigation (e.g. colonoscopy)
- Day hospital (e.g. Medicine for the Elderly)
- Physiotherapy
- Occupational therapy
- Speech and language therapy
- Psychology / counselling services
- Respite care
- Pharmacist (apart from collecting your tablets)
- Other: _____________________________________________________________________________

Has the COVID-19 Pandemic impacted on your access to any of the services above, and if so how?

- Service no longer available
- Service suspended or postponed
- Service postponed as I had a procedure/appointment in a private hospital/practice
- I’m on a waiting list for this service
- Other______________________________________________________________________________

## **Section D: General Questions**

**Q1** What is your current civil status? *Civil status, or marital status, are the distinct options that describe a person's relationship with a significant other. Please select one option*

- Partner
- Married
- Separated
- Divorced
- Widowed
- Never married/single

**Q2** Who do you currently live with? *Please select all that apply*

- Husband/wife/partner
- Family/relatives
- Flat/house share
- Live alone
- Other

**Q3** What best describes your present principal status? *Please select one option*

- Employee (incl. community employment)
- Self-employed without employees
- Self-employed with employees
- Looking after home/family
- Retired from employment
- Unemployed
- Student

**Q4** What is the highest level of education you have completed to date? *Please select one option*

- No formal education
- Primary education
- Secondary education
- Technical or vocational qualification: trade apprentice, certificate or diploma or equivalent
- Third level education

**Q5** Did the COVID-19 pandemic impact on your decision to attend today? YES/NO

*If yes, please select all that apply*

- I delayed my attendance
- I was more cautious about attending
- I wasn’t sure what to expect in the ED
- I consulted with a healthcare professional before attending for advice
- I consulted with a friend/family member for advice before attending
- Other, please specify ___________________________________________________________________________________

**Q6** If you were concerned about attending due to COVID-19, on a scale of 1-10 (10 being most concerned) how would you rate your level of concern? ___________________________________________________________

**THANK YOU FOR PARTICIPATING**
