# Supplementary material for: The impact of COVID-19 on an Irish Emergency Department (ED): a cross-sectional study exploring the factors influencing ED utilisation prior to and during the pandemic from the patient perspective
Source: BMC Emerg Med. 2022 Nov 2;22:176. doi: 10.1186/s12873-022-00720-7 (PMC9628103; doi:10.1186/s12873-022-00720-7)
Supplement: Supplementary file 2 — Additional file 2: Table S1. General Practitioner Consultation prior to Attendance at the ED (n=174) a. [file 12873_2022_720_MOESM2_ESM.docx]

**Additional File 2 – Supplementary Table S1**

**Table S1. General Practitioner Consultation prior to Attendance at the ED (n=174)** *^a^*

| **Did you consult your GP prior to ED Attendance?** | **TOTAL** | **DEC** | **FEB** | **JUL** | **P value** |
| --- | --- | --- | --- | --- | --- |
| Yes, I saw my GP and was told to go to the ED | 51% | 45% | 57% | 50% | 0.504 |
| Yes, I had a phone consultation with my GP and was sent to ED | 13% | 6% | 11% | 19% | 0.127 |
| Yes, I saw my GP but was unhappy with the treatment | 1% | 2% | 0% | 0% | 0.257 |
| Yes, I tried but could not contact my GP | 4% | 2% | 5% | 3% | 0.643 |
| Yes, but GP was unable to treat due to COVID-19 restrictions | - | - | - | 10% | - |
| No, I thought that my problem is best dealt with in the ED | 15% | 17% | 20% | 7% | 0.107 |
| No, the GP surgery was closed | 6% | 13% | 0% | 7% | ≤0.05 |
| No, I thought my GP would refer me to the ED | 2% | 2% | 2% | 1% | 0.960 |
| No, the GP is further away than the ED | 1% | 2% | 0% | 0% | 0.257 |
| No, I did not want to bother my GP | 1% | 0% | 0% | 1% | 0.474 |
| No, I do not have a GP | 1% | 2% | 0% | 0% | 0.257 |
| No, I am not happy with my current GP | 0% | 0% | 0% | 0% | 0.916 |
| No, some other reason   - Referral from other service - Ambulance/Immediate Care required - Miscellaneous | 20%  8%  5%  7% | 21%  11%  6%  4% | 21%  7%  5%  9% | 19%  7%  4%  7% | 0.494 |

*^a^ Individual cases of missing data were excluded from analysis, this occurred in a small number of cases E.g. Data was not traceable on hospital systems or in the event a participant close not to respond to a question on the survey.*
