# Supplementary material for: The impact of COVID-19 on an Irish Emergency Department (ED): a cross-sectional study exploring the factors influencing ED utilisation prior to and during the pandemic from the patient perspective
Source: BMC Emerg Med. 2022 Nov 2;22:176. doi: 10.1186/s12873-022-00720-7 (PMC9628103; doi:10.1186/s12873-022-00720-7)
Supplement: Supplementary file 3 — Additional file 3: Table S2. Health Service Utilisation in the last 12 months (n=174) a. [file 12873_2022_720_MOESM3_ESM.docx]

**Additional File 3 – Supplementary Table S2**

**Table S2. Health Service Utilisation in the last 12 months (n=174)** *^a^*

| **Health Service** | **Frequency** | **TOTAL**  **(n=174)** | **DEC**  **(n=47)** | **FEB**  **(n=57)** | **JUL**  **(n=70)** | **P value** |
| --- | --- | --- | --- | --- | --- | --- |
| **Hospital Admission** | 0  1  2-3  4-6  7+ | 113, 65%  35, 20%  19, 11%  3, 2%  4, 2% | 34, 72%  7, 15%  3, 6%  2, 4%  1, 2% | 38, 67%  14, 25%  3, 5%  0, 0%  2, 4% | 41, 59%  14, 20%  13, 19%  1, 1%  1, 1% | p=0.262 |
| **Out-Patient Department** | 0  1  2-3  4-6  7+ | 97, 56%  25, 14%  41, 24%  8, 5%  3, 2% | 26, 55%  4, 9%  11, 23%  5, 11%  1, 2% | 29, 51%  10, 18%  16, 28%  2, 4%  0, 0% | 42, 60%  11, 16%  14, 20%  1, 1%  2, 3% | p=0.503 |
| **Emergency Department** | 0  1  2-3  4-6  7+ | 79, 45%  47, 27%  39, 22%  4, 2%  5, 3% | 24, 51%  10, 21%  10, 21%  0, 0%  3, 6% | 26, 46%  17, 30%  12, 21%  1, 2%  1, 2% | 29, 41%  20, 29%  17, 24%  3, 4%  1, 1% | p=0.705 |
| **Injury Unit** | 0  1  2-3  4-6  7+ | 158, 91%  11, 6%  4, 2%  0, 0%  0, 0% | 43, 92%  3, 6%  1, 2%  0, 0%  0, 0% | 50, 88%  5, 9%  2, 4%  0, 0%  0, 0% | 65, 94%  3, 4%  1, 1%  0, 0%  0, 0% | p=0.436 |
| **Out-of-Hours GP** | 0  1  2-3  4-6  7+ | 125, 72%  28, 16%  18, 10%  0, 0%  2, 1% | 30, 64%  10, 21%  6, 13%  0, 0%  1, 2% | 43, 75%  8, 14%  6, 11%  0, 0%  0, 0% | 52, 75%  10, 15%  6, 9%  0, 0%  1, 1% | p=0.329 |
| **General Practitioner** | 0  1  2-3  4-6  7+ | 14, 8%  29, 17%  67, 39%  26, 15%  38, 22% | 5, 11%  5, 11%  16, 34%  7, 15%  14, 30% | 4, 7%  8, 14%  26, 46%  8, 14%  11, 19% | 5, 7%  16, 23%  25, 36%  11, 16%  13, 19% | p=0.429 |
| **Public Health Nurse** | 0  1  2-3  4-6  7+ | 147, 85%  4, 2%  12, 7%  7, 4%  4, 2% | 38, 81%  2, 4%  4, 9%  1, 2%  2, 4% | 48, 84%  1, 2%  5, 9%  2, 4%  1, 2% | 61, 87%  1, 1%  3, 4%  4, 6%  1, 1% | p=0.694 |
| **Allied Health Professional*** | 0  1  2-3  4-6  7+ | 139, 80%  8, 5%  11, 6%  11, 6%  5, 3% | 37, 79%  2,4%  2,4%  3, 6%  3, 6% | 45, 79%  1, 2%  4, 7%  6, 11%  1, 2% | 57, 81%  5, 7%  5, 7%  2, 3%  1, 1% | p=0.798 |
| **Other Services and Additional Details** | | | | | | |
| **Health Service** | **Frequency** | **TOTAL** | **DEC** | **FEB** | **JUL** | **P value** |
| Hospital Day Case | ≥1 | 21% | 30% | 12% | 23% | 0.087 |
| Day Hospital Consult | ≥1 | 5% | 11% | 4% | 3% | 0.139 |
| Physiotherapy | ≥1 | 10% | 11% | 14% | 7% | 0.446 |
| Occupational Therapy | ≥1 | 3% | 4% | 2% | 3% | 0.749 |
| Speech & Language | ≥1 | 1% | 0% | 2% | 0% | 0.356 |
| Psychology/Counselling | ≥1 | 3% | 2% | 0% | 7% | 0.076 |
| Respite Care | ≥1 | 1% | 0% | 2% | 1% | 0.678 |
| Pharmacy | ≥1 | 9% | 11% | 7% | 10% | 0.781 |

*^a^ Individual cases of missing data were excluded from analysis, this occurred in a small number of cases E.g. Data was not traceable on hospital systems or in the event a participant close not to respond to a question on the survey.*
